# The first-in-human preclinical evaluation of the new probe [123I]I-PSMA-7 for real-time intraoperative targeted biopsy and SPECT/CT imaging in prostate cancer

**DOI:** 10.1007/s00259-024-06833-4

**Published:** 2024-07-23

**Authors:** Xiaohui Luan, Shaoxi Niu, Yachao Liu, Xiaojun Zhang, Xiaodan Xu, Shuwei Sun, Yabing Sun, Jingfeng Zhang, Yuan Wang, Zhiqiang Chen, Yimin Chen, Mengchao Cui, Ruimin Wang, Xu Zhang, Jinming Zhang, Baixuan Xu

**Affiliations:** 1 Department of Nuclear Medicine, The First Medical Centre of Chinese PLA General Hospital, Beijing, China; 2 Chinese PLA General Hospital, Chinese PLA Medical School, Beijing, China; 3 Department of Urology, The Third Medical Centre of Chinese PLA General Hospital, Beijing, China; 4 Key Laboratory of Radiopharmaceuticals, Ministry of Education, College of Chemistry, Beijing Normal University, Beijing, China

**Keywords:** [^123^I]I-PSMA-7, Real-time targeted biopsy, PSMA SPECT/CT imaging, Clinical study

## Abstract

**Purpose:**

PSMA/PET has been increasingly used to detect PCa, and PSMA/PET-guided biopsy has shown promising results. However, it cannot be confirmed immediately whether the tissues are the targeted area. In this study, we aimed to develop a novel probe, [^123^I]I-PSMA-7. First, we hope that [^123^I]I-PSMA-7 can provide instant confirmation for prostate biopsy. Second, we hope it will help detect PCa.

**Methods:**

We synthesized a high-affinity probe, [^123^I]I-PSMA-7, and evaluated its properties. We included ten patients with suspected PCa and divided them into two groups. The injection and biopsy were approximately 24 h apart. The activity in biopsy lesions was measured as the cpm by a γ-counter. Moreover, we enrolled 3 patients to evaluate the potential of [^123^I]I-PSMA-7 for detecting PCa.

**Results:**

Animal experiments verified the safety, targeting and effectiveness of [^123^I]I-PSMA-7, and the tumor-to-muscle ratio was greatest at 24 h, which confirmed the results of this study in humans. After injection of 185MBq [^123^I]I-PSMA-7, 18/55 cores were positive, and the cpm was significantly greater (4345 ± 3547 vs. 714 ± 547, *P* < 0.001), with an AUC of 0.97 and a cutoff of 1312 (sens/spec of 94.40%/91.90%). At a lower dose, 10/55 biopsy cores were cancerous, and the cpm was 2446 ± 1622 vs. 153 ± 112 (*P* < 0.001). The AUC was 1, with a cutoff value of 490 (sens/spec of 100%). When the radiopharmaceuticals were added to 370 MBq, we achieved better SPECT/CT imaging.

**Conclusion:**

With the aid of [^123^I]I-PSMA-7 and via cpm-based biopsy, we can reduce the number of biopsies to a minimum operation. [^123^I]I-PSMA-7 PSMA SPECT/CT can also provide good imaging results.

**Trial registration:**

Chinese Clinical trial registry ChiCTR2300069745, Registered 24 March 2023

## Introduction

Prostate cancer (PCa) is one of the leading causes of cancer death in men worldwide [1]. Accurate diagnosis of PCa is crucial for patients to choose treatments, such as surgery, radiation therapy and immunotherapy [2]. Prostate-specific membrane antigen (PSMA) positron emission tomography (PET) has resulted in the increasing detection of PCa [3]. However, prostate biopsy (PB) remains a mandatory step for confirming PCa, although almost 25% of patients will experience complication such as lower urinary tract symptoms [4]. The sensitivity of transrectal ultrasound (TRUS) of PB is only approximately 50% [5]. Multiparametric MRI (mpMRI) is recommended for prostate cancer diagnosis, and MRI-guided transrectal ultrasound (MR-TRUS) biopsy increases the detection of clinically significant PCa (csPCa) [6]. MR-TRUS is usually used in patients with a high clinical suspicion of PCa despite a negative systematic biopsy [7]. One study confirmed that [^68^Ga]Ga-PSMA PET/CT combined with PET/ultrasound-guided prostate biopsy, can effectively detect csPCa [8]. Another study used [^68^Ga]Ga-PSMA-11 PET/MRI-guided biopsy in patients with suspected prostate cancer and the sensitivity was 96% [9]. With the increasing use of upfront diagnostic MRI and PSMA PET, these biopsies are expected to replace routine systematic biopsies.

Although mpMRI or PSMA PET provide a precise location of prostate cancer, for example, [^18^F]F-PSMA-1007 is excreted through the liver and can avoid the influence of urine retention in the bladder on the detection of lesions [10], they provide only graphic support. With only imaging support, the surgeon cannot determine whether the biopsy tissue is prostate cancer in real time, so to avoid missing the lesion, the doctor will still choose the standard 12-needle biopsy [11]. As a result, even under the guidance of high-quality images, the patient’s burden during biopsy is not reduced, and the resulting inflammation will delay the time to undergo surgery [12]. If radionuclides are still present at the time of the patient’s biopsy, we can use the radionuclide count to determine whether we have reached the high metabolic site immediately. The most commonly used ^18^F- and ^68^Ga-labeled PSMA have short half-lives and are expensive. It is not practical for every patient to undergo this examination before prostate biopsy. To maximize patient benefits, we developed a new probe, [^123^I]I-PSMA-7. We evaluated the ability of [^123^I]I-PSMA-7 to guide real-time targeted prostate biopsy and the diagnostic performance for PCa.

## Materials and methods

### General materials, cell lines and mouse models

Analytical grade reagents, solvents and chemicals purchased from Maclin Biochemical Technology Co., Ltd. (Shanghai, China) were used for synthesis and analysis. Materials such as T_25_ cell culture flasks and trypsin were obtained from Gibco Life Technologies (Grand Island, NY, USA).

The 22Rv1 and PC3 cell lines were obtained from GuYan Biotech Co., Ltd. (Shanghai, China). The cells were cultured in RPMI-1640 medium supplemented with 1% penicillin/streptomycin and 10% fetal bovine serum (FBS) (Gibco Life Technologies, Grand Island, NY, USA). All cells were cultivated in an incubator containing 5% CO_2_ at 37 °C and grown to 80–90% confluence before trypsinization.

All animal experiments conformed to the protocol approved by the Animal Care and Use Committee of the PLA General Hospital. BALB/c male nude mice (approximately 3–4 weeks old) weighing 13–15 g were purchased from Charles River Laboratories (Beijing, China). Approximately 5 × 10^6^ cells were implanted into the right shoulder of each mouse. Mice were imaged or used in biodistribution assays when the tumor volume reached 200–300 mm^3^. The same implantation method was used for PC3 as for 22Rv1.

### Chemical synthesis, radiolabeling and quality control

The chemical synthesis and radiolabeling of [^123^I]I-PSMA-7 were identical to those of [^125^I]I-PSMA-7, with only a change between the isotopes of iodine [13]. Radio-HPLC was used to determine the purity of [^123^I]I-PSMA-7. The mobile phases were 75% solvent A (H_2_O) and 25% solvent B (acetonitrile + 0.1% TFA) and the flow rate was set to 1 mL/min. [^123^I]I-PSMA-7 solution was added to 5% phosphate-buffered saline (PBS) or a bovine serum albumin (BSA) solution. After 1 h, 2 h, 4 h, 6 h, 8 h, 24 h at room temperature or 37 °C, radio-HPLC was used to determine the radiochemical purity. The experiment was repeated three times per time point.

### Acute toxicity test, ex vivo biodistribution and imaging

[^123^I]I-PSMA-7 (74 MBq in 100 µL) or saline was injected into healthy male ICR mice, that were 3–4 weeks old and weighed 20–25 g. Then, the diet, activity, mental state, skin, and other indicators of the two groups were observed daily. On the 14th day, the mice were sacrificed and the main organs were removed to observe whether there were significant differences between the two groups in terms of color, shape, texture, and other aspects. The organs were stained with HE.

To evaluate the distribution of [^123^I]I-PSMA-7 in 22Rv1 tumor-bearing mice, we carried out biological distribution experiments. Twenty-one 22Rv1 tumor-bearing mice were randomly divided into seven groups (*n* = 3/group). [^123^I]I-PSMA-7 solution (5.55 × 10^4^ Bq, 100 µL) was injected at 1 h, 2 h, 4 h, 6 h, 8 h, 24 h and 48 h. The tumors and major tissues and organs (blood, urine, heart, lung, liver, spleen, kidney, bladder, tumor, brain, muscle, bone, salivary glands, and small intestine) were collected at the prescribed times. The radioactive count of the tissues and organs was measured by a γ-counter after they were washed and dried with normal saline and weighed using an electronic balance and the corresponding radioactive value was calculated after time attenuation correction (%ID/g).

22Rv1 and PC3 tumor-bearing mice were subjected to SPECT/CT (3D whole-body scan, MMP919 collimator, TriFoil console-gr157 Triumph, America) imaging studies, reconstructed with HiSPECT software (California, America) and analyzed by Vivoquant 2.5 software (Osaka, Japan). Three 22Rv1 tumor-bearing mice were randomly selected for injection of [^123^I]I-PSMA-7 (37 MBq, 100 µL). The mice were placed in an anesthesia box and a 3.0% isoflurane/air gas mixture was diffused into the box before the prescribed time. The mice exhibited no voluntary activity after 5 min and were placed in a prone position on the scanning bed and maintained using a 1.0% isoflurane/air gas mixture during the scanning.

In blocking experiments, 22Rv1 tumor-bearing mice were generated 60 min after coinjection of ZJ43 (50 mg/kg) with [^123^I]I-PSMA-7 (37 MBq, 100 µL) [14]. SPECT images of the PC3 tumor-bearing mice were used as a negative control.

### Study design of real-time targeted biopsy for prostate cancer

The study was designed as a single-center, randomized, prospective study and conformed to the protocol approved by the Ethics Committee of the Chinese PLA General Hospital (approval of No. S2022-576 Registration number of ChiCTR2311119745). The inclusion and exclusion criteria were as follows: patients aged 40 to 85 years, whose PSA level was persistently elevated (> 4 ng/mL) or whose imaging findings indicated the presence of suspicious lesions; and patients who underwent biopsy and whose pathology results were available in the hospital. The exclusion criteria included patients who had serious underlying disease, who accepted androgen deprivation hormonal therapy (ADT) or who had previous pelvic irradiation. All patients signed a dedicated informed consent form (Table 1). All patients received Lugol’s iodine for more than 24 h before the injection of [^123^I]I-PSMA-7 to protect the thyroid gland. Five patients (Patients 1–5) were injected with [^123^I]I-PSMA-7 at 185 MBq and then underwent a SPECT/CT scan. To maximize the possibility of promoting real-time targeted technology, this study did not rely on PSMA-PET imaging; the suspicious lesions indicated by MRI are also significant; therefore, another 5 patients (Patients 6–10) were injected with only 55 MBq radiopharmaceuticals at a lower dose and without SPECT/CT imaging. The time interval between injection and biopsy was approximately 24 h.

Before the biopsy, exposure rates were measured at 10 cm and 1 m from the patient to ensure safety in the operating room. Biopsies and pelvic local anesthesia were performed via ultrasound (BK5000, Denmark). We carried out US-guided perineal prostate biopsy, with the first needle pointing to the target area, followed by standard 12-needle biopsy.

After the biopsy, the samples were transferred immediately to a gamma-counter to obtain the cpm (Wiper PHILIPS-05-500). The measurement time was 1 min, and the energy window was set from 0 to 1000 KeV. Each biopsy sample was placed in a microcentrifuge tube to obtain the cpm value, after which the tissue was transferred to formalin for histopathological assessment. The counts were correlated with histopathology, ISUP grade, and tumor volume.

### Preliminary clinical SPECT/CT imaging in humans

The preliminary study enrolled 3 patients (Table 1, Patients 11–13). They were injected with [^123^I]I-PSMA-7 (370 MBq, 0.5 mL) and subjected to a whole SPECT/CT scan (GE Discovery NM/CT 670 CZT); the time interval between the scans was approximately 4 h. The parameters of the SPECT scan were as follows: low-energy high-resolution collimator, a matrix of 256 × 256, and a scan speed of 5 cm/min. The CT scan parameters were as follows: a matrix of 512 × 512, 200 KeV, 140 mA, and 5 mm slice thickness. The images were analyzed by 2 experienced physicians.

### Statistical analysis

SPSS 27.0 (New York, America) and GraphPad Prism 9.0 (San Diego, America.) *P* < 0.05 was considered to indicate statistical significance. Normally distributed data are expressed as the mean ± standard deviation and were analyzed via independent-samples t tests. The median and quartile spacing, homogeneity of variance and Wilcoxon rank sum tests were performed on the data that were not normally distributed. The cutoff for distinguishing between positive and negative tissues was the receiver operating characteristic (ROC) curve and the area under the ROC curve (AUC).

## Results

### Quality control, radiotoxicity, biodistribution, and micro-SPECT imaging

**Fig. 1:**
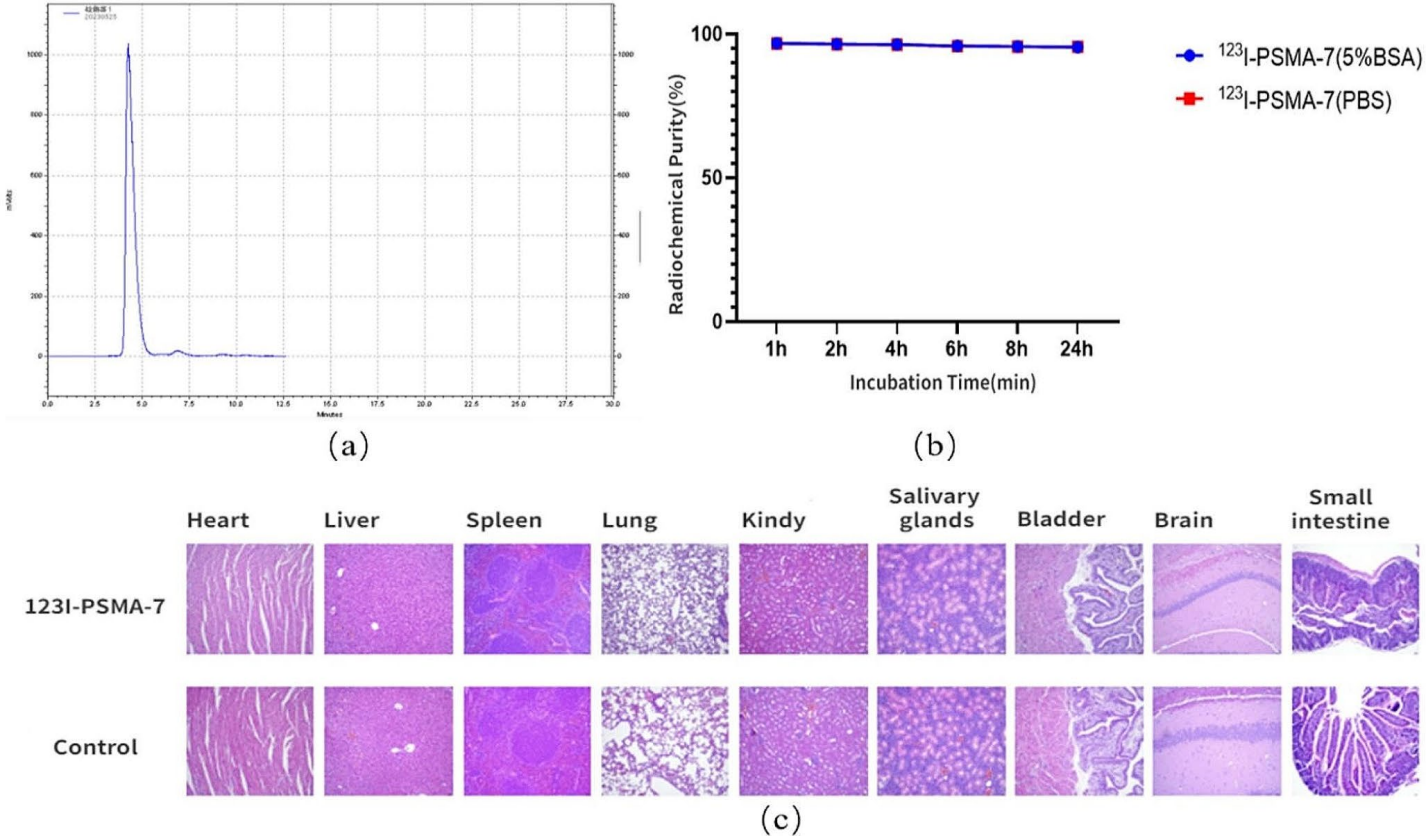
(**a**) HPLC chromatograms of [^123^I]I-PSMA-7. (**b**) In vitro stability of [^123^I]I-PSMA-7 at 1 h, 2 h, 4 h, 6 h, 8 h, and 24 h at room temperature or 37 °C. (**c**) The results of acute toxicity tests

**Fig. 2:**
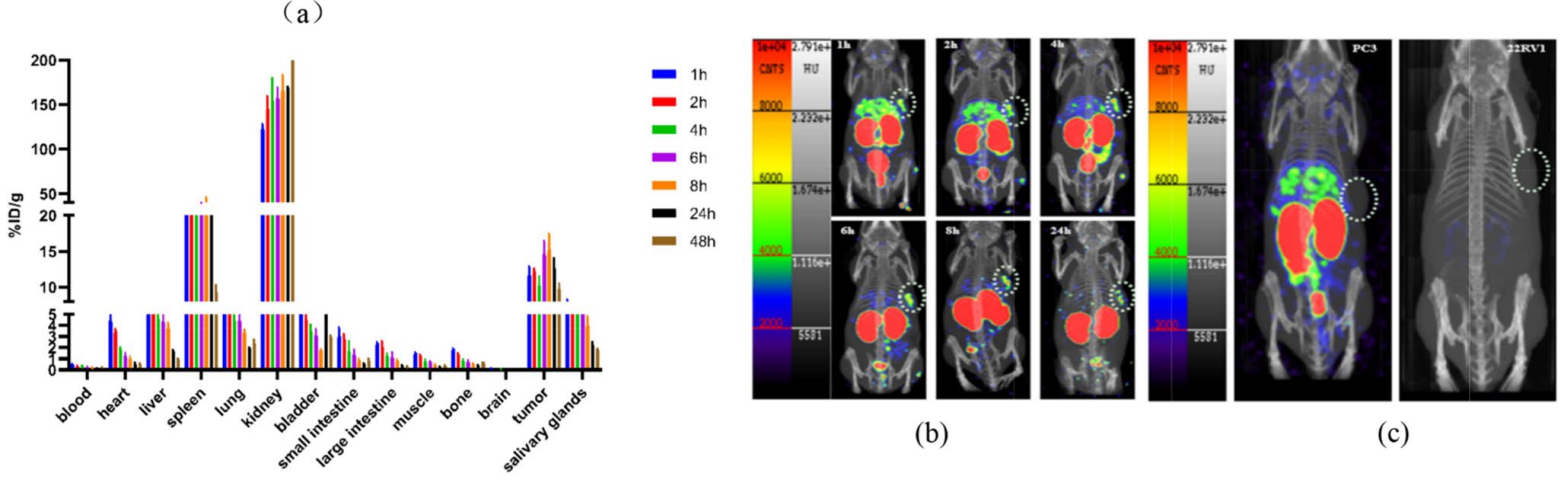
(**a**) The distribution of [^123^I]I-PSMA-7 in 22Rv1 tumor-bearing mice. (**b**) SPECT/CT images of [^123^I]I-PSMA-7 in 22Rv1 tumor-bearing mice. (**c**) SPECT/CT imaging of PC3 tumor-bearing mice and 22Rv1 tumor-bearing mice coinjected with ZJ43. In PC3 tumor-bearing mice, the tumor could not be observed, while the kidneys were clearly visible

The purity of [^123^I]I-PSMA-7 remained greater than 95% for 24 h (Fig. 1a, b). During the acute toxicity test, the HE staining of the experimental group was the same as that of the control group, both of which were normal (Fig. 1c). Over the 2 days, the maximum uptake of [^123^I]I-PSMA-7 in the tumor was 15.24 ± 2.25% ID/g at 8 h. The maximum tumor-to-muscle ratio was approximately 50 at 24 h. The organs with high uptake were the kidneys, salivary glands and spleen (Fig. 2a). After 24 h, the accumulation of [^123^I]I-PSMA-7 in the tumors of the 22Rv1 tumor-bearing mice was observed. In contrast, in PC3 tumor-bearing mice and 22Rv1 tumor-bearing mice coinjected with ZJ43, the tumors could not be visualized (Fig. 2b, c).

**Table 1:**
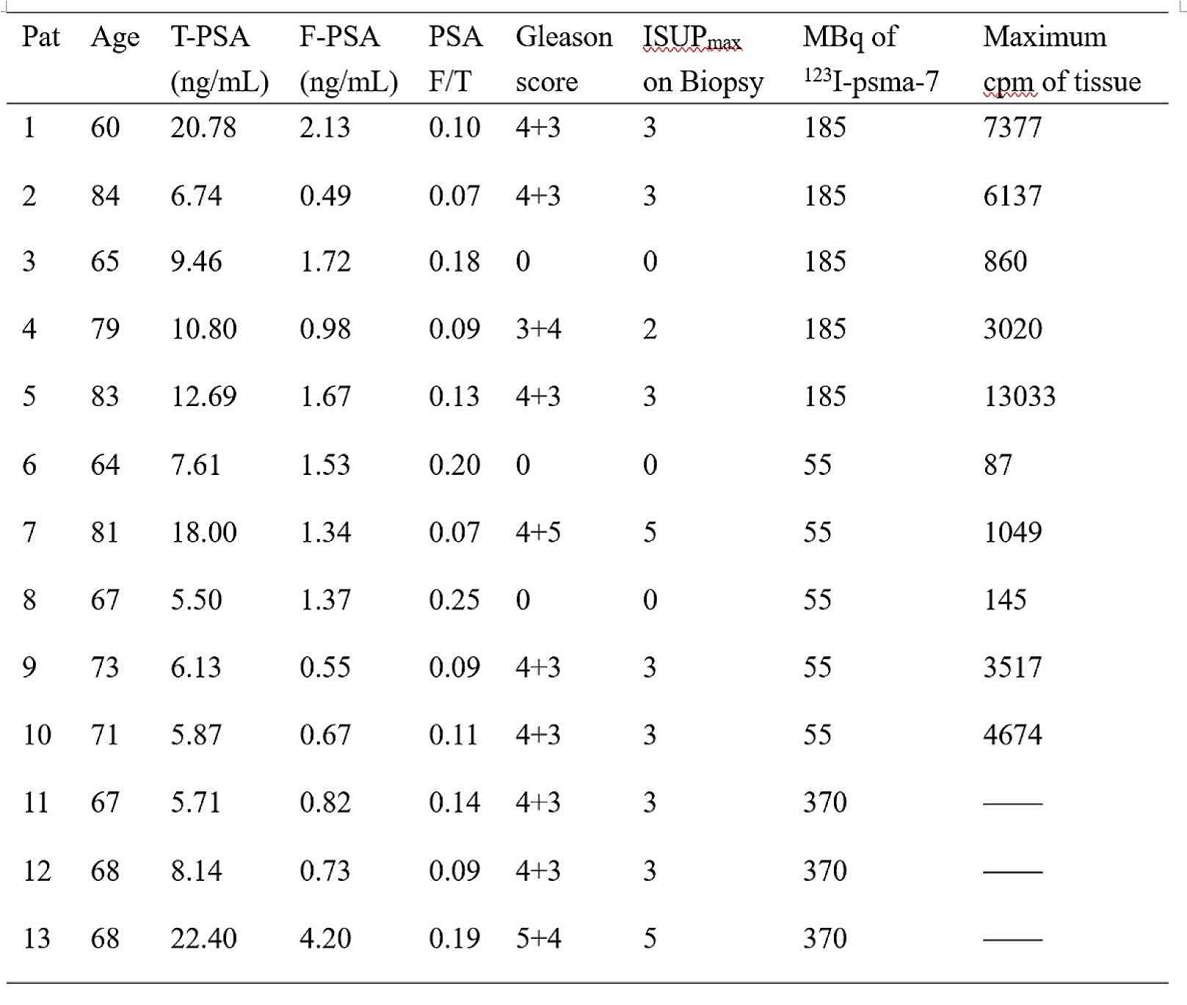
Individual patient characteristics and the CPM of prostate cancer patients

### Analysis of real-time targeted biopsy results after treatment with 185 MBq [^123^I]I-PSMA-7

**Fig. 3:**
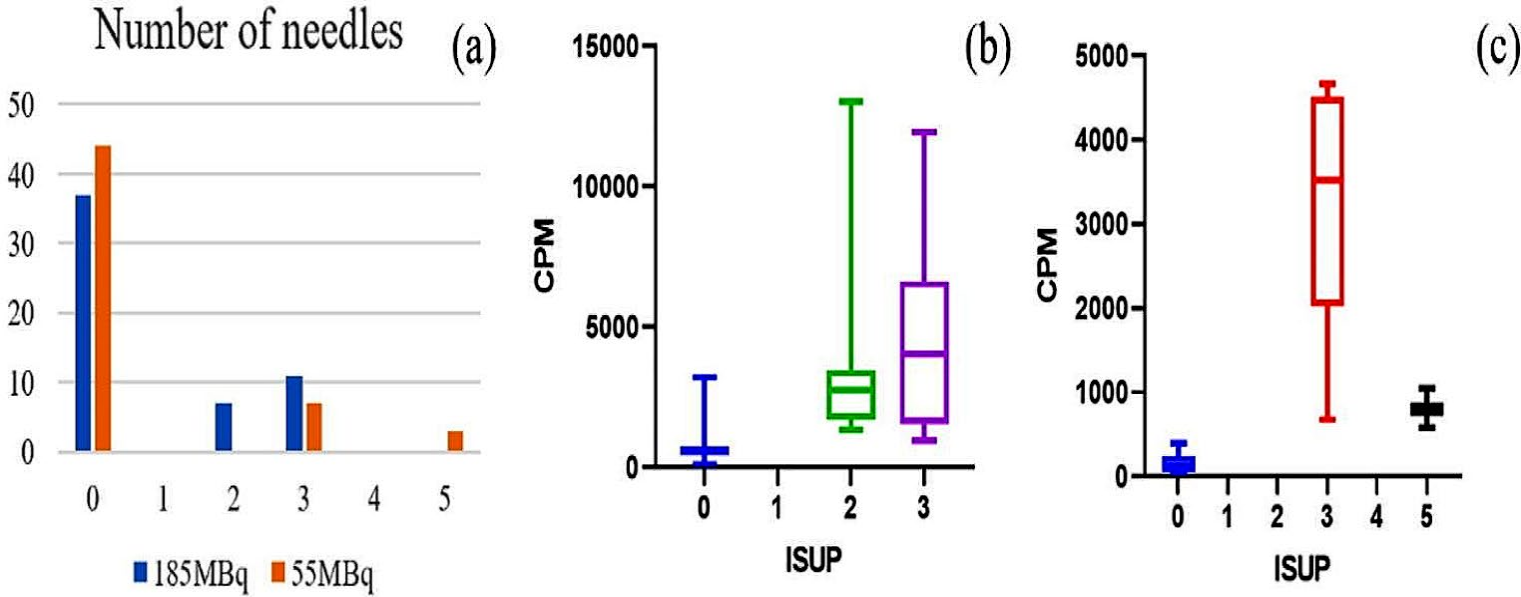
(**a**) The number of needles for each biopsy ISUP grade of 185 MBq and 55 MBq. (**b**) Box plot for the cpm distribution according to the biopsy ISUP grade of 185 MBq. (**c**) Box plot for the cpm distribution according to the biopsy ISUP grade of 55 MBq

**Fig. 4:**
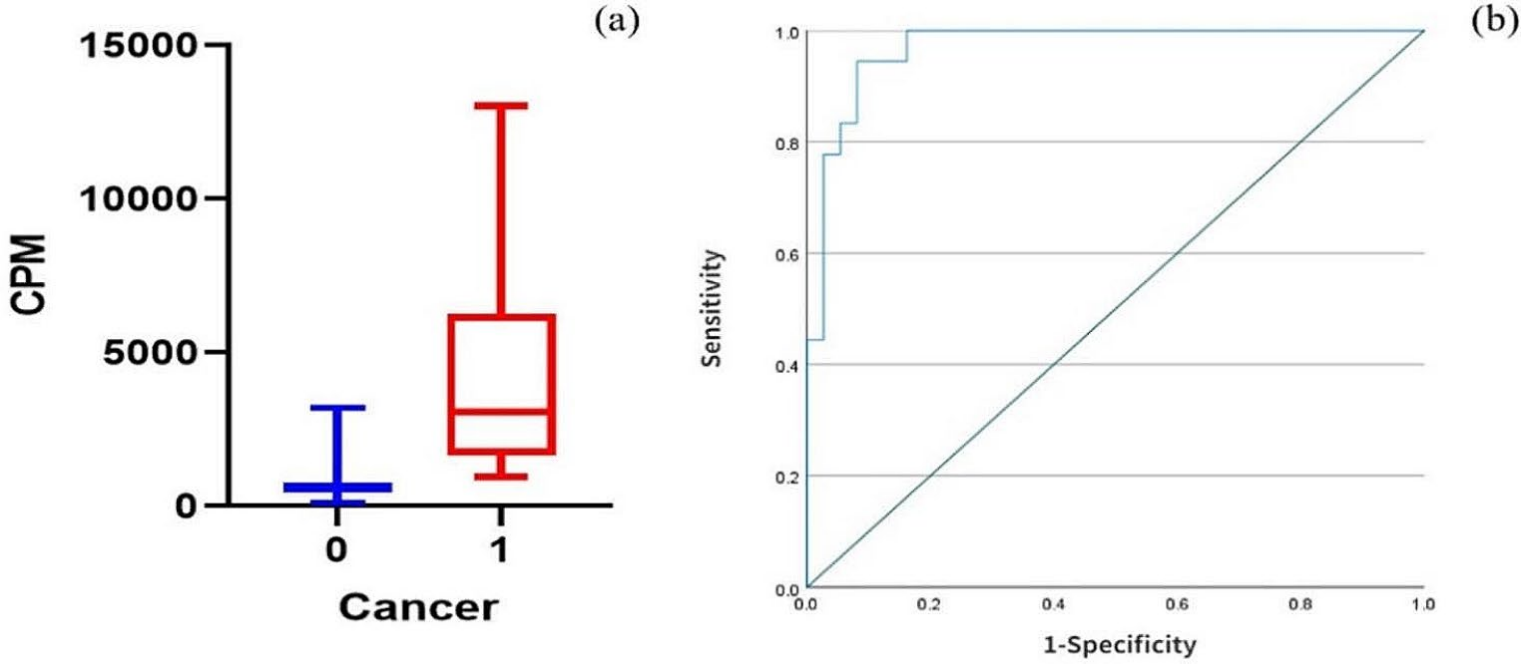
(**a**) Box plot for the cpm distribution according to the absence (0) or presence (1) of PCa after injecting 185 MBq [^123^I]I-PSMA-7. (**b**) The ROC curve of 185-MBq [^123^I]I-PSMA-7-injected patients

In this study, we included 5 patients (age, 72.40 ± 10.99 years, range 60–84 years). Table 1 shows the characteristics of the patients. In 4 of 5 patients (80%), sigPCa was observed with a maximum ISUP of 2 or 3 (Fig. 3). Pathology revealed that 18 of the 55 cores were positive for PCa. The cpm was significantly greater in PCa patients than in normal controls (4345 ± 3547 vs. 714 ± 547, *P* < 0.001). To assess the value of the cpm for intraoperatively detecting PCa, we obtained an AUC of 0.97, with a cutoff of 1312 and a sensitivity and specificity of 94.40% and 91.90%, respectively (Fig. 4). A total of 35/55 biopsies were fewer than 1312, and 1/35 were false-negativenegatives. A total of 20/55 cpm more than 1312 and 3/20 were false-positivepositives according to histopathology.

**Fig. 5:**
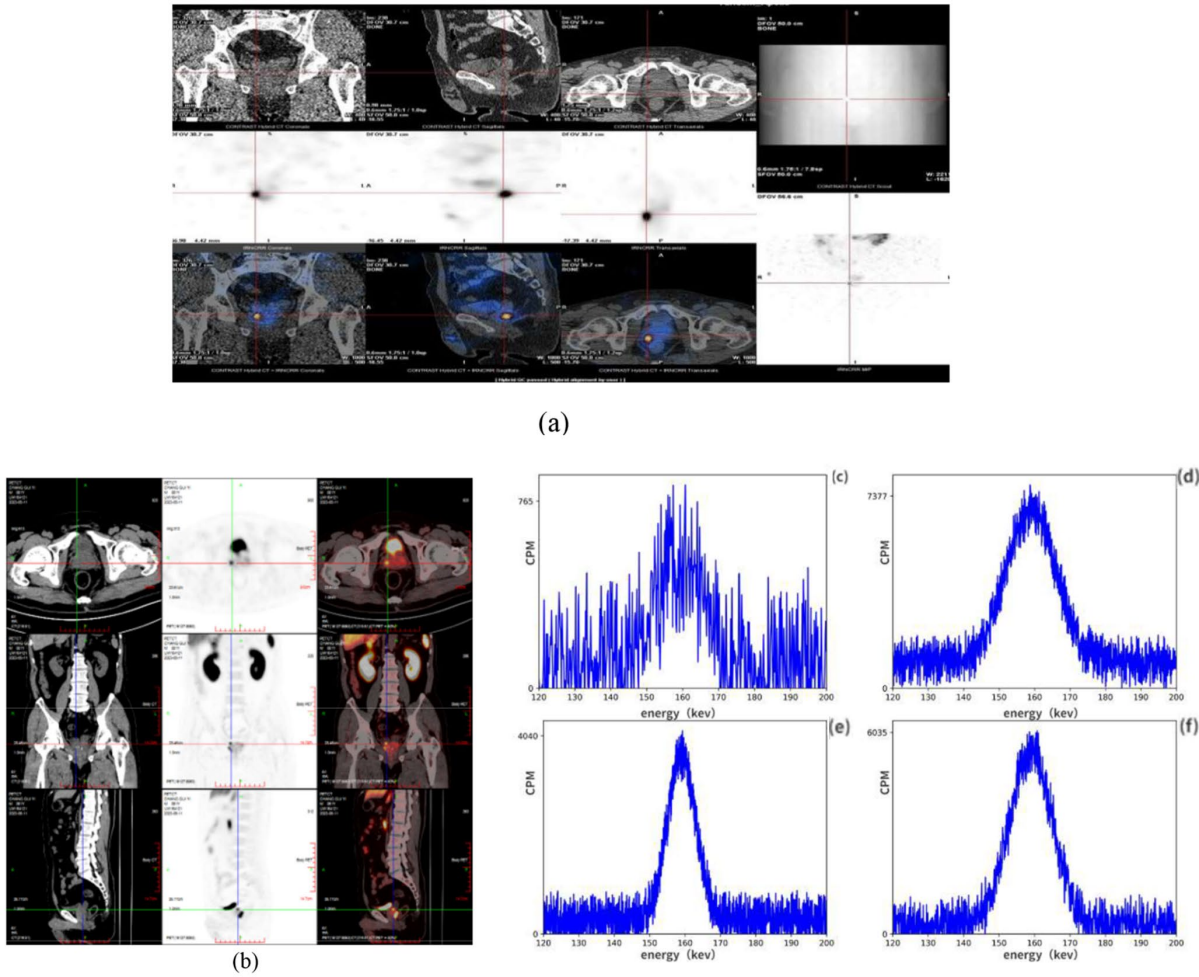
(**a**–**b**) Imaging by [^123^I]I-PSMA-7 SPECT/CT and [^68^Ga]Ga-PSMA-11 PSMA/PET in Patient 1. (**c**) Negative tissue from Patient 1. (**d**–**f**) The positive tissues of Patient 1

For Patient 1, we obtained 24 h point [^123^I]I-PSMA-7 SPECT/CT images to observe the intake of the radiopharmaceuticals. The lesion was clearly visible on the image and was basically consistent with the [^68^Ga]Ga-PSMA-11 PET/CT imaging. The highest cpm of patient 1 was 7677, approximately 10 times greater than that of the negative control tissue (Fig. 5). The exposure rate was measured at 10 cm as < 2 µSv/h, and the exposure rate at 1 m was close to the environmental background.

### The search for lower doses of [^123^I]I-PSMA-7 for real-time targeted biopsy

**Fig. 6:**
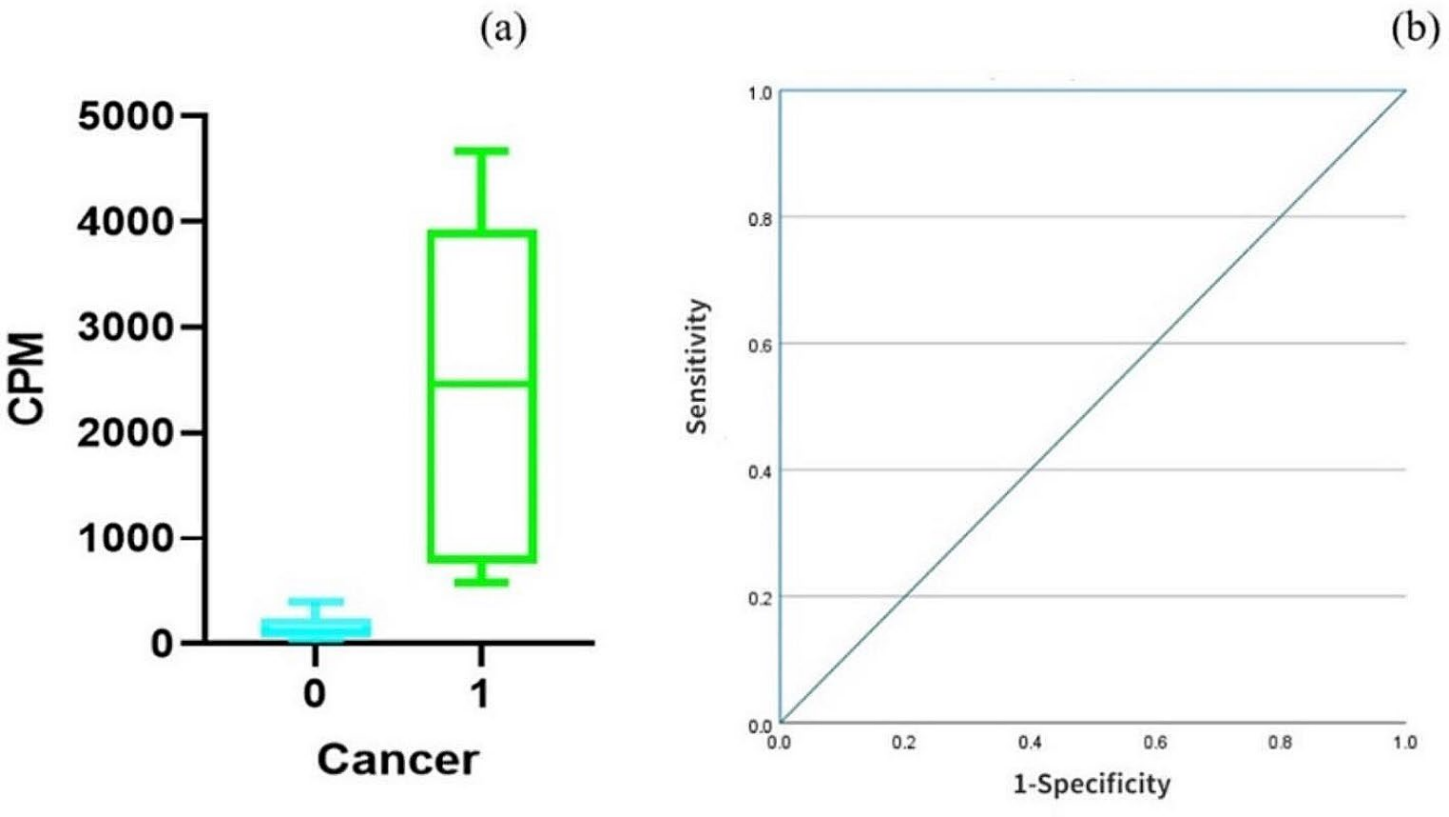
(**a**) Box plot for the cpm distribution according to the absence (0) or presence (1) of PCa after injecting 55 MBq [^123^I]I-PSMA-7. (**b**) The ROC curve of patients injected with 55 MBq [^123^I]I-PSMA-7

We included five patients aged between 64 and 81 years (age, 71.20 ± 6.50 years). Table 1 presents the patient demographics. In 60% of these patients (3 out of 5 patients), significant prostate cancer (sigPCa) was detected, reaching an ISUP grade of 3 or 5 at the highest (Fig. 3). Pathology revealed that 10 out of 55 biopsy cores were cancerous. We found a significant difference in the cpm between cancerous and noncancerous cores (2446 ± 1622 vs. 153 ± 112, *P* < 0.001). To evaluate the effectiveness of the cpm for detecting PCa intraoperatively, we calculated an area under the curve (AUC) of 1. With a cutoff value of 490, we achieved a sensitivity of 100% and a specificity of 100% (Fig. 6).

**Fig. 7:**
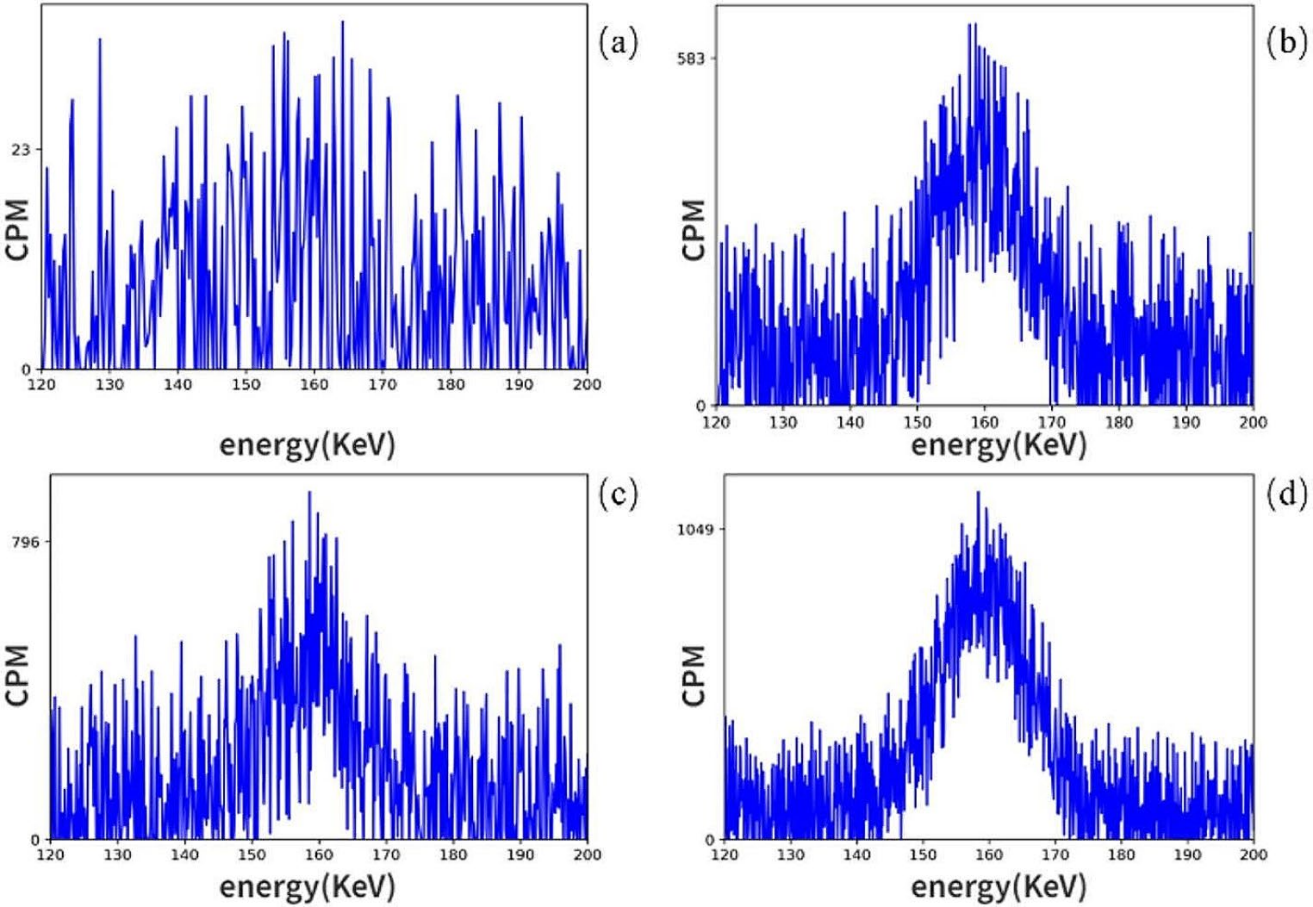
(**a**) After the background counter, the first no-loading count is approximately 20 cpm. (**b**–**d**) The positive tissues of Patient 7. There were 23 negative tissue samples, but the number of positive tissue samples was nearly 25 times greater

Figure 7 shows the cpm of one patient. There were 23 negative tissue samples, but the number of positive tissue samples was nearly 25 times greater. Additionally, we measured the exposure rate at 10 cm to be less than 0.5 µSv/h, while at 1 m, it was nearly equivalent to environmental background levels.

### The value of SPECT/CT imaging in prostate cancer

**Fig. 8:**
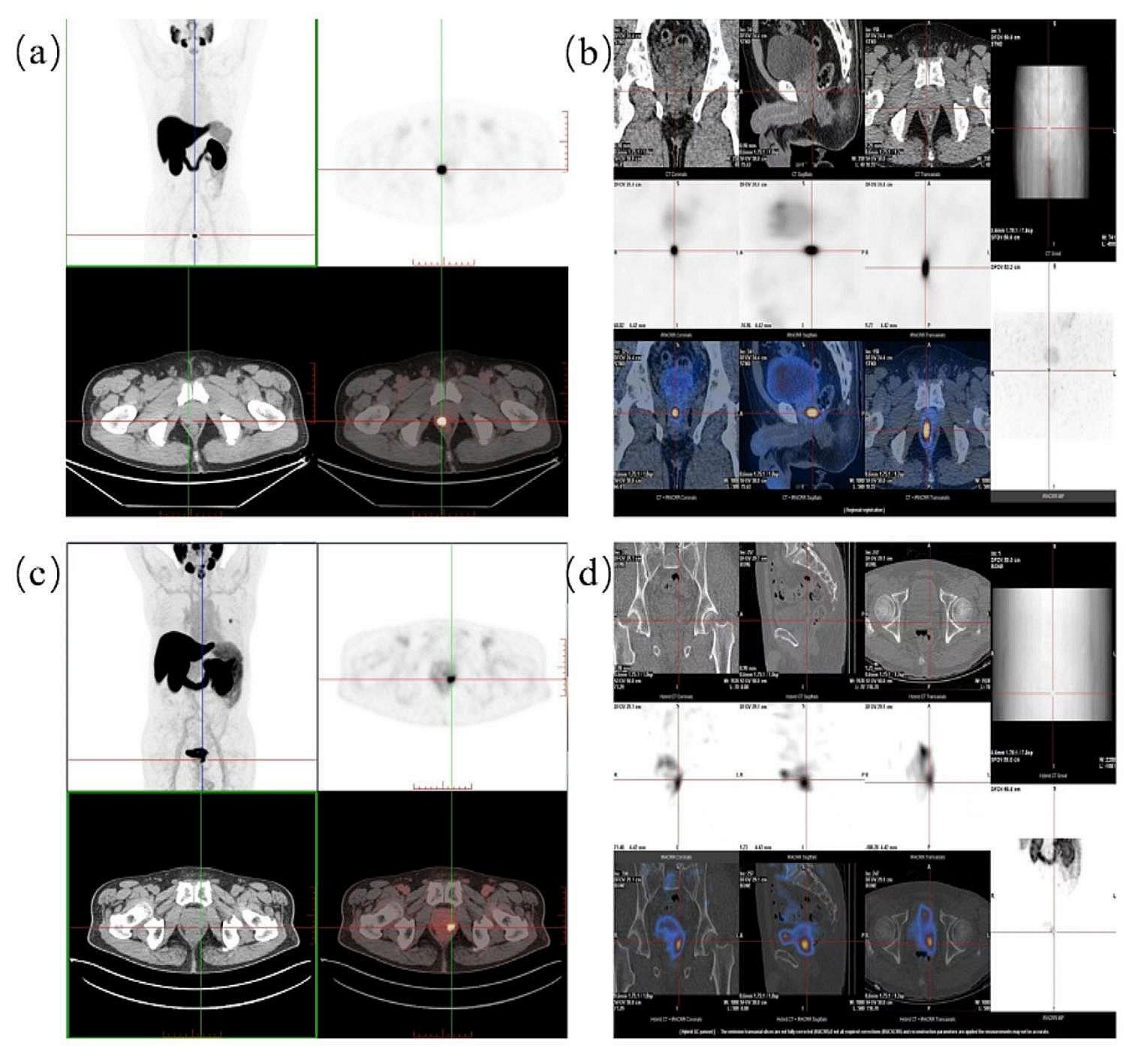
(**a**) Imaging of [^18^F]F-PSMA-7Q in Patient 11. (**b**) Imaging of [^123^I]I-PSMA-7 in Patient 11 at 4 h after injection. (**c**) Imaging of [^18^F]F-PSMA-7Q in Patient 12. (**d**) Imaging of [^123^I]I-PSMA-7 in Patient 12 at 4 h after injection

To evaluate the value of [^123^I]I-PSMA-7 SPECT/CT for detecting PCa lesions, we added radiopharmaceuticals to 370 MBq to obtain better images. The patients also underwent [^18^F]F-PSMA-7Q-PSMA PET/CT. A picture of patient 11, 67 years old, with a PSA concentration of 5.71 ng/mL, and patient 12, 68 years old, with a PSA concentration of 8.14 ng/mL, is shown in Fig. 8. The lesion was clearly visible on the [^123^I]I-PSMA-7 SPECT/CT image and was basically consistent with the [^18^F]F-PSMA-7Q PET/CT image.

## Discussion

For localizing the foci of prostate cancer, PSMA PET outperforms other imaging techniques [15–18]. Prostate biopsy can also be performed depending on the indication for imaging. Growing evidence has proven the efficacy of mpMRI and PSMA PET/CT-guided targeted biopsy (TB) in PCa diagnosis. One study showed that dual-tracer (gastrin-releasing peptide receptor GRPR and PSMA) positron emission tomography (PET)/CT-TB plus systematic biopsy might be a more effective and promising strategy for the definite diagnosis of csPCa than mpMRI-TB [19]. Ultrasound fusion biopsy targeted by [^18^F]F-DCFPyL PET/MRI is an identical pathway for the detection of prostate cancer [20]. However, imaging cannot reveal whether the tissues are prostate cancer in real time. Real-time biopsy has been performed in a few studies. We believe that there are two reasons for this: PET is not widespread, and the positron developer has a short half-life. Therefore, we synthesized a novel radiopharmaceutical, [^123^I]I-PSMA-7, to overcome the above problems. The tumor uptake and tumor muscle ratio in early animal experiments provided confidence for prostate cancer diagnostic imaging and real-time targeted biopsy experiments.

Initially, we wanted to inject 185 MBq [^123^I]I-PSMA-7 to achieve a balance between prostate imaging and real-time biopsy, and the time interval between them was 24 h to ensure the safety of radiation. A dose of 185 MBq [^123^I]I-PSMA-7 could detect lesions, but the image quality was poor, and patient 1 had new lesions on [^68^Ga]Ga-PSMA-11 PET/CT. In addition, due to patient 1’s intolerance to the pain, only a few targeted biopsy needles were used, but this did not affect the results. Overall, the ability of 185 MBq [^123^I]I-PSMA-7 to guide real-time biopsy is excellent. According to the cutoff of 1312, 4/55 was false in total. In addition, we found that the proportion of tumors, tumor uptake, etc., are influencing factors. However, we want to minimize the impact of these factors, and we pursue the feasibility of real-time judgment. We analyzed the data and obtained an area under the curve (AUC) of 0.97 and a sensitivity and specificity greater than 90%. Real-time targeted biopsy can be performed, but the image quality still needs to be improved. Because a small number of people receive PSMA PET before biopsy, most people receive MRI [21]. Next, we explored higher doses to obtain better image quality and lower doses to explore the possibility of real-time targeted biopsy independent of PSMA-guided images. A lower dose, 55 MBq, of [^123^I]I-PSMA-7 eliminates the dependence on PSMA PET and has the highest realization and promotion potential. The results of low-dose experiments showed better results; as far as the current data are concerned, 185 MBq was satisfactory. When the injection dose reached 370 MBq, the quality of the PSMA SPECT/CT was better than that before, and no obvious lesions were missed compared with the PSMA PET/CT.

PSMA-1007 is mainly excreted through the liver, and this metabolic pathway rules out the interference of urine in the bladder, which can more effectively identify the primary cause of prostate cancer. This technique was used in a previous study to guide the biopsy [22]. In that study, [^18^F]F-PSMA-1007 was used to detect radioactivity in tissues instead of ^68^Ga because of its longer half-life. They included five patients with suspected PCa who underwent [^18^F]F-PSMA-1007 PET/CT scans followed by immediate PET/CT-guided saturation template biopsy. Then, the activity in the biopsy cores was measured. Excluding the patient with moderate PSMA, ROC analysis revealed an AUC of 0.81, with an optimal cutoff to confirm PCa at 75 cpm (sens/spec of 65.1%/87%). The time window of biopsy after imaging is too narrow, which is not conducive to clinical practice. In the preliminary experiment, we verified the potential of [^125^I]I-PSMA-7 for real-time targeted biopsy, but considering the visualized imaging, we changed it to [^123^I]I-PSMA-7. With the same precursor, human imaging results confirmed the possibility of using [^125^I]I-PSMA-7 for long-term window biopsy. At the same time, we will also focus on the application of new technologies and probes. We also have independent intellectual property rights for [^18^F]F-PSMA-7Q, which is mainly excreted through the liver [23]. Robot-assisted biopsy is safe and has a high diagnostic yield for PCa for PSMA-avid lesions [24]. One study showed that [^68^Ga]Ga-PSMA CLI (Cerenkov luminescence imaging) is a promising and safe technique for intraoperative margin assessment in PCa patients, and it may also help achieve real-time targeted biopsy [25]. At present, [^123^I]I-PSMA-7 has achieved satisfactory results for imaging and real-time biopsy, and we will expand the caseload in the future.

## Conclusion

With cpm-based biopsy, we can confirm accurate prostate cancer tissue operatively and have the possibility to reduce biopsy to a minimum. [^123^I]I-PSMA-7 PSMA SPECT/CT can also provide good PCa imaging when the dose reaches 370 MBq.

## Data Availability

Data is contained within the article.

## References

[1] Bray F, et al. Global cancer statistics 2022: GLOBOCAN estimates of incidence and mortality worldwide for 36 cancers in 185 countries. CA Cancer J Clin. 2024;74(3):229–63. 38572751 10.3322/caac.21834

[2] Schaeffer EM, Cancer P, et al. Version 4.2023, NCCN Clinical Practice guidelines in Oncology. J Natl Compr Canc Netw. 2023;21(10):1067–96. 37856213 10.6004/jnccn.2023.0050

[3] Wu Q, et al. Detection rate of PSMA PET using different ligands in men with biochemical recurrent prostate Cancer following radical treatment: a systematic review and ,meta-analysis of prospective studies. Acad Radiol. 2024;31(2):544–63. 37770370 10.1016/j.acra.2023.08.044

[4] Borghesi M, et al. Complications after systematic, Random, and image-guided prostate biopsy. Eur Urol. 2017;71(3):353–65. 27543165 10.1016/j.eururo.2016.08.004

[5] Brock M, et al. Detecting prostate cancer. Dtsch Arztebl Int. 2015;112(37):605–11. 26396046 10.3238/arztebl.2015.0605 PMC4581108

[6] Mason BR, et al. Current status of MRI and PET in the NCCN guidelines for prostate Cancer. J Natl Compr Canc Netw. 2019;17(5):506–13. 31085758 10.6004/jnccn.2019.7306

[7] Das CJ, et al. MRI-Targeted prostate biopsy: what radiologists should know. Korean J Radiol. 2020;21(9):1087–94. 32691544 10.3348/kjr.2019.0817 PMC7371617

[8] Liu C, et al. (68)Ga-PSMA PET/CT combined with PET/Ultrasound-Guided prostate biopsy can diagnose clinically significant prostate Cancer in men with previous negative biopsy results. J Nucl Med. 2020;61(9):1314–9. 32034111 10.2967/jnumed.119.235333 PMC7456174

[9] Ferraro DA, et al. Diagnostic performance of (68)Ga-PSMA-11 PET/MRI-guided biopsy in patients with suspected prostate cancer: a prospective single-center study. Eur J Nucl Med Mol Imaging. 2021;48(10):3315–24. 33620559 10.1007/s00259-021-05261-y PMC8426229

[10] Privé BM, et al. Evaluating F-18-PSMA-1007-PET in primary prostate cancer and comparing it to multi-parametric MRI and histopathology. Prostate Cancer Prostatic Dis. 2021;24(2):423–30. 32999466 10.1038/s41391-020-00292-2

[11] Dieffenbacher S, et al. Standardized magnetic resonance imaging reporting using the prostate Cancer Radiological estimation of change in sequential evaluation criteria and Magnetic Resonance Imaging/Transrectal Ultrasound Fusion with Transperineal Saturation Biopsy to select men on active surveillance. Eur Urol Focus. 2021;7(1):102–10. 30878348 10.1016/j.euf.2019.03.001

[12] Liu Y, et al. A pilot study of (18)F-DCFPyL PET/CT or PET/MRI and Ultrasound Fusion targeted prostate biopsy for intra-prostatic PET-Positive lesions. Front Oncol. 2021;11:612157. 33747927 10.3389/fonc.2021.612157 PMC7973269

[13] Luan X, et al. A preclinical study of an (125)I-Labeled PSMA Ligand for prostate-Cancer puncture. Pharmaceuticals (Basel). 2022; 15(10). 10.3390/ph15101252 PMC9610460 36297363

[14] Liu T, et al. Development of an albumin-based PSMA Probe with prolonged half-life. Front Mol Biosci. 2020;7:585024. 33392253 10.3389/fmolb.2020.585024 PMC7773938

[15] Gandaglia G, et al. Should we combine systematic with MRI-targeted biopsy? Implications for the management of patients with prostate cancer. Eur Radiol. 2022;32(11):7488–90. 36107203 10.1007/s00330-022-09096-5

[16] Chen Y et al. Synthesis, preclinical evaluation, and first-in-human PET study of [(68)Ga]-Labeled biphenyl-containing PSMA Tracers. J Med Chem. 2023. 10.1021/acs.jmedchem.3c01475 37708404

[17] Liu Y, et al. Comparison between (18) F-DCFPyL PET and MRI for the detection of transition zone prostate cancer. Prostate. 2021;81(16):1329–36. 34516670 10.1002/pros.24230

[18] Liu Y, et al. Prospective intraindividual comparison of 18F-PSMA-7Q and 18F-DCFPyL PET/CT in patients with newly diagnosed prostate cancer. Nucl Med Commun. 2022;43(6):725–30. 35560134 10.1097/MNM.0000000000001564

[19] Qiu DX, et al. Dual-tracer PET/CT-targeted, mpMRI-targeted, systematic biopsy, and combined biopsy for the diagnosis of prostate cancer: a pilot study. Eur J Nucl Med Mol Imaging. 2022;49(8):2821–32. 34860277 10.1007/s00259-021-05636-1

[20] Niu S et al. (18) F-DCFPyL positron emission tomography/magnetic resonance imaging-guided ultrasound fusion biopsy is an identical pathway in prostate cancer diagnosis. Prostate. 2023;83(2): 142–150. 10.1002/pros.24446 36281654

[21] Wang X, et al. A prospective multi-center randomized comparative trial evaluating outcomes of transrectal ultrasound (TRUS)-guided 12-core systematic biopsy, mpMRI-targeted 12-core biopsy, and artificial intelligence ultrasound of prostate (AIUSP) 6-core targeted biopsy for prostate cancer diagnosis. World J Urol. 2023;41(3):653–62. 35852595 10.1007/s00345-022-04086-0

[22] Ferraro DA, et al. Hot needles can confirm accurate lesion sampling intraoperatively using [(18)F]PSMA-1007 PET/CT-guided biopsy in patients with suspected prostate cancer. Eur J Nucl Med Mol Imaging. 2022;49(5):1721–30. 34725726 10.1007/s00259-021-05599-3 PMC8560591

[23] Zhang X, et al. Synthesis, preclinical evaluation, and first-in-human PET study of Quinoline-Containing PSMA tracers with decreased renal excretion. J Med Chem. 2021;64(7):4179–95. 33783213 10.1021/acs.jmedchem.1c00117

[24] Kumar R, et al. Safety and Diagnostic Yield of (68)Ga prostate-specific membrane antigen PET/CT-guided robotic-assisted transgluteal prostatic biopsy. Radiology. 2022;303(2):392–8. 35191735 10.1148/radiol.204066

[25] Olde Heuvel J, et al. (68)Ga-PSMA Cerenkov luminescence imaging in primary prostate cancer: first-in-man series. Eur J Nucl Med Mol Imaging. 2020;47(11):2624–32. 32242253 10.1007/s00259-020-04783-1 PMC7515945

